# Stakeholder survey about broad elements of value in health technology assessment in Australia: industry and academia more similar than different

**DOI:** 10.1017/S0266462325100226

**Published:** 2025-07-08

**Authors:** Maria Farris, Stephen Goodall, Richard De Abreu Lourenco

**Affiliations:** 1 Astra Zeneca, Health Economics, Macquarie Park, NSW, Australia; 2 Centre for Health Economic Research and Evaluation, University of Technology Sydney, Ultimo, NSW, Australia

**Keywords:** rare diseases, uncertainty, advisory committees, technology assessment, biomedical

## Abstract

**Objective:**

Researchers propose wider individual and societal benefits (or broad elements of value) be included in economic evaluations (EEs) of medicines. This study investigates opinions of Australian stakeholders regarding the inclusion of broader value elements in reimbursement decisions for medicines for rare diseases in Australia.

**Method:**

Stakeholders were invited via email to complete an online survey about their views on broader elements of value in HTA. Responses were summarised using descriptive statistics and compared using chi-square statistics.

**Results:**

Forty-four respondents (academia (n=11), private sector (n=33)) completed the survey between October 2023 and May 2024. Only 27% of stakeholders agree the current information about the sources of value considered in reimbursement decisions is sufficient. Stakeholders consistently agree labour productivity (>50%), adherence (>80%), reducing uncertainty due to a new diagnostic (>70%), disease severity (>71%), value to caregivers (>70%), and equity (>70%) should be considered in HTA. The majority (>70%) agreed managed entry agreements (MEA), risk share arrangements (RSA), and multi criteria decision analysis (MCDA) be used in reimbursement decision making for medicines for rare diseases. Significantly fewer academic stakeholders (40%) versus private sector (77%), believe an increased willingness-to-pay threshold be applied to medicines for rare disease.

**Conclusions:**

Academic and private sector stakeholders hold similar views when considering medicines for non-rare and rare diseases. Stakeholders favour considering more value elements in HTA than referred to in the Pharmaceutical Benefits Advisory Committee (PBAC) guidelines. This study highlights further advice is needed on the factors considered in reimbursement decisions and how that would influence guidelines.

## Introduction

Economic evaluation (EE) is widely used in health technology assessment (HTA) to inform reimbursement decisions in healthcare (1;2). As part of HTA, an EE assesses the incremental cost-effectiveness (CE) of a new therapy, and the incremental CE ratio (ICER) is judged against an implicit or explicit “CE threshold,” to help judge the efficient allocation of healthcare resources (3).

EEs can only include benefits for which adequate data are generated (4). Typically, only direct patient health benefits via quality of life (QoL) and survival (used to calculate quality-adjusted life years) are considered in an EE (5). However, they can adopt a wider, societal perspective and incorporate broader elements of value, such as indirect nonhealth benefits offered by a medicine (6–8). The perspective taken by decision makers is often outlined by HTA guidelines reflecting their country’s values and preferences, and they may be required to consider a government perspective only, rather than a societal perspective (1;5).

Several studies suggest that wider benefits to individuals and society should be included in EEs (9–11). An International Society for Pharmacoeconomics and Outcomes Research (ISPOR) special task force on value assessment recommends a series of broader value elements in HTA assessments (9). Because if HTA does not include the broader value of a therapy, then treatments with wide-ranging impacts may be undervalued and receive inappropriately high ICERs (8;12). Some of the broad value elements suggested range from conventional concepts, such as adherence-improving factors or disease severity, to novel elements of value, such as scientific spillover (9).

Rare diseases are a group of diverse diseases, characterized by low prevalence and often have severely debilitating symptoms that substantially affect the QoL of patients and their families (13;14). EEs of medicines for rare diseases often produce high and uncertain ICERs, in part due to their high cost and difficulty generating robust evidence supporting clinical efficacy due to small sample sizes, single-arm studies, shorter duration of patient follow-up, and reliance on immature clinical evidence to inform modeling (3;13). Different reimbursement agencies provide varying recommendations based on EEs of the same medicine for rare disease, partly because factors like disease severity and indirect treatment benefits were considered, leading to greater acceptance of higher and uncertain ICERs (15;16). Additionally, some rare disease medicines have gained expedited access in cases of high unmet need through payment mechanisms, such as outcome-based managed entry agreements (MEAs), to address the financial risks associated with uncertain clinical evidence (17).

To improve the quality of EEs, experts recommend an impact inventory to explicitly consider the broader health and nonhealth impacts of a medicine (7). Because methods to include broad value elements into value assessment are unclear, HTA agencies use different approaches (18–20). Two mechanisms to formally include broader elements of value into an EE are a multi-criteria decision analysis (MCDA) or the deliberative process (21). The latter is used by reimbursement agencies, such as the National Institute for Health and Care Excellence (NICE) in England and Wales and the Pharmaceutical Benefits Advisory Committee (PBAC) in Australia. However, deliberative processes have their shortcomings, as the relative importance of various criteria varies among stakeholders and the elements of value contributing to the decision are not always clear, and how the decision was reached is not always transparent (13;21–25).

This study investigates the opinions of Australian stakeholders in the HTA process about the importance of various broader elements of value in EEs, transparency in reimbursement decision making in Australia, and opinions on mechanisms to manage uncertainty associated with medicines for rare diseases.

## Methods

A quantitative survey was conducted of stakeholders involved in HTA in Australia, representing academia, specialist consultants, and the pharmaceutical industry. An invitation was emailed to potential participants (including government agencies and representatives of patient organizations) via local professional societies, and invitees were encouraged to forward the survey to other relevant colleagues. No responses were received from government agencies or patient representatives.

The survey was developed using the Qualtrics Survey platform and was completed between 2 October 2023 and 14 May 2024. Questions were based on the broader elements of value proposed by ISPOR and the mechanisms suggested, and adopted, to manage uncertainty in value assessment (9;23;24;26). The questions were discussed with an expert health economist experienced in HTA before implementation. Before the initiation of the survey, the appropriateness and order of the questions were discussed within the research team. Pilot testing of the survey was conducted with internal and external members of the research team to assess comprehension. The survey comprised thirty-two questions across six sections (Supplement S1) and was intended to take ~10 minutes to complete.

Because value elements are sometimes referred to by other names in the literature or the names may not represent the essence of what is considered, a brief description of each “value element” was included in the survey. A description of the broader elements of value and mechanisms to manage uncertainty in value assessment, as presented in the survey, is provided in Table 1.

**Table 1.** Description of the broader elements of value and mechanisms to manage uncertainty in value assessment presented in the survey

Broader element of value
**Labor productivity:** *Relates to costs associated with production loss and replacement costs due to illness, disability, and death of productive persons, both paid and unpaid.*
**Adherence**: *Patient adherence and health outcomes relating to advantageous, simpler dosing schedules, alternate routes of administration, or combination treatments over existing alternatives.*
**Reducing uncertainty due to a new diagnostic**: *A companion diagnostic test that could differentiate “good responders” and “poor responders” may provide the ability to avoid an ineffective treatment in poor responders, as well as costs and consequences of treatment-related adverse events.*
**Fear of contagion:** *Reducing the anxiety associated with the risk of future illness, even if the expected number of cases prevented is low.*
**Insurance value**: *Reflects the value of an effective treatment for a disease, reducing fear among all consumers of getting the disease.*
**Severity of disease**: *A gain in health may be more valuable to patients with a poor baseline prognosis (i.e., more severe disease).*
**Value of hope**: *Reflects the extent to which the chance for a cure is valued. For example, some patients may be willing to trade some survival (e.g., undertake a risky procedure) for a chance of a “cure” even if only for a small probability of cure/improved survival.*
**Value to caregiver**: *The extent to which health care can benefit family carers by reducing their caring responsibilities.*
**Real option value:** *Value generated when a health technology that extends life creates opportunities for the patient to benefit from other future advances in medicine.*
**Scientific spillovers**: *Broad societal benefit from knowledge created from a treatment with a new mechanism of action. It is considered a public good used for the discovery of other agents.*
**Equity:** *Fairness in the distribution of health and health care within society, across rich and poor, young and old, marginalized and not, employed or unemployed, for example.*

Mechanisms to manage uncertainty in value assessment
**Multi-criteria decision analysis (MCDA**): *A deliberative process where decision makers and stakeholders define the problem and determine the criteria, weighting, and evidence requirements for a reimbursement decision.*
**Willingness to pay (WTP):** *Increase the ICER considered acceptable for treatments of rare diseases.*
**Outcome-based managed entry agreements (MEAs):** *Allows earlier market access but requires CEA review once additional outcome data are available. For example, clinical data from prespecified study protocol for all patients subsidized or from existing planned or progressing studies.*
**Financial risk-sharing arrangements (RSAs):** *Financial subsidy based on medicine or patient performance. For example, percentage rebate if the number of treatments per patient or the accepted duration of treatment is exceeded. Subsidy ceases if patients do not meet agreed clinical measures.*

Most questions sought agreement to statements on a 5-point Likert scale: 1 = *strongly disagree*, 2 = *somewhat disagree*, 3 = *neither disagree nor agree*, 4 = *somewhat agree*, 5 = *strongly agree* (respondents could choose a sixth category “Don’t know”). Depending on the resulting number of respondents, and to ensure >5 minimum responses per category (for statistical testing), the categories “strongly agree” and “somewhat agree” were collapsed into one group (“Agree”), and the categories “strongly disagree” and “somewhat disagree” into another group (“Disagree”). The category “neither disagree nor agree” or “don’t know” is henceforth referred to as “Neither” within the text. The remaining questions asked participants whether they agreed with statements with response options “yes,” “no,” or “not sure,” and to nominate methods (via a free text field) to incorporate added value not currently utilized in EEs. If the response was “yes,” the participant was reported to “Agree.” A response of “no” or “not sure” reflected that the participant did not agree with the statement.

Five major categories of stakeholders were defined for respondents to self-allocate: (i) pharmaceutical industry, (ii) specialist consultants, (iii) academia, (iv) government agency, and (v) representative of patient organization. Within the Australian HTA process, academia is responsible for the independent evaluation of reimbursement applications. Specialist consultants, typically HTA consultancy firms, are engaged by the pharmaceutical industry to assist in the preparation of these applications. Responses to each question were summarized using descriptive statistics and reported for the cohort overall and by respondent categories separately. Tests for differences between respondent categories were performed using *χ* ^2^-statistics (5 percent significance level). The relative risk (RR) (academic group vs. private sector groups) and 95 percent confidence interval (CI) are estimated for each response.

Where no background demographics were reported for a participant who consented, their data were removed from the sample. If demographic data were reported but only partial survey response data were provided, participant responses were only included in those questions to which they contributed (thus, the sample size varies per question).

All analyses were performed using Excel on an MS Windows platform.

This study received ethics approval in September 2023 (HREC REF No. ETH21-6090).

## Results

Forty-four respondents completed the survey from academia (*n* = 11) and the private sector (*n* = 33). The respondent categories were aggregated into “academia” and the “private sector” (pharmaceutical industry and specialist consultants). The sample was adjusted by excluding three respondents without demographic data. The majority of respondents in both groups had a postgraduate degree (Masters 27/44, 61 percent, or Doctoral 14/44, 32 percent), and the top three primary qualifications were in health economics (28/44, 64 percent), pharmacy (11/44, 25 percent), and science (10/44, 23 percent). The mean (standard deviation) years of experience were 7.3 years in academia and 14.3 years in the private sector (Table 2). Most respondents (67 percent) in the private sector held managerial roles compared with only 18 percent (2/11) of respondents in academic group.

**Table 2.** Background information for all stakeholders and by subgroup

	Australian stakeholders (*N* = 44)	Australian stakeholder subgroups	
		Academia (*N* = 11)	Private (consultants = 10, pharmaceutical industry = 23)
Years involved in HTA in Australia, mean	12.1 years	7.3 years	14.3 years
Position Managerial, *n*/*N* (%)	22/44 (50%)	2/11 (18%)	20/33 (61%)
Academic qualification, *n*/*N* (%)	14/44 (32%) Doctoral degree 27/44 (61%) Master’s degree 3/44 (7%) Undergraduate degree	8/11 (72%) Doctoral degree 3/11 (27%) Master’s degree	6/33 (18%) Doctoral degree 24/33 (72%) Master’s degree 3/33 (10%) Undergraduate degree
Area of academic qualification,^a^ *n*/*N* (%)	28/44 (64%) Health economics 11/44 (25%) Pharmacy 6/44 (14%) Statistics 10/44 (23%) Science 6/44 (14%) Public health 7/44 (16%) Business/Economics/MBA	9/11 (82%) Health economics 2/11 (18%) Statistics 1/11 (9%) Science 1/11 (9%) Pharmacy 1/11 (9%) Public health	19/33 (58%) health economics 10/33 (30%) Pharmacy 9/33 (27%) Science 7/33 (21%) Business/Economics/MBA 4/33 (12%) Statistics 1/33 (<1%) Medicine 5/33 (15%) Public heath

a Multiple disciplines reported per individual in some cases.

Few (<30 percent) Australian stakeholders agree that the current HTA methods applied in Australia are adequate to appropriately assess the CE of all medicines, including medicines for rare diseases (Table 3). Despite the absence of a significant difference in responses between stakeholders, it is noteworthy that academic respondents were four times more likely (RR = 4.36) to agree that the HTA methods used in Australia are adequate for all medicines, compared to their private sector counterparts. However, the substantial uncertainty surrounding this estimate is reflected in the wide CI (range = 0.84–22.79). The majority of stakeholders disagreed with the statement that the public information on reimbursement decisions in Australia provides sufficient information about which sources of value are considered and how they contribute to decision making (73 percent; academia = 55 percent vs. private sector = 80 percent, *p* = .1031) (Table 3). It is important to emphasize the variation in response rates, despite the lack of statistical significance. Notably, academics were twice as likely (RR = 2.27) to concur that the publicly available information on reimbursement decisions is adequate compared with the private sector. Of the twenty-four respondents from the private sector who disagreed, 83 percent felt that while they knew which sources of value were considered, they did not know how they contributed to decision making. Equal proportions of respondents from academia thought that either the sources of value considered were not known (33 percent) or they did not know how they contributed to decision making (33 percent).

**Table 3.** Comparison between adequacy of HTA methods, sufficiency of public information, and mechanisms for decision making

Q: Do you think the current HTA methods applied in Australia are adequate to appropriately assess the cost-effectiveness of all medicines? *n*/*N* (%) agree			
Total cohort	5/43 (12%)		
Academia	3/11 (27%)	RR: 4.36 (95% CI = .84, 22.79), *p* = .06	
Private sector	2/32 (6%)		
Q: Do you think the current HTA methods applied in Australia are adequate to appropriately assess the cost-effectiveness of medicines for rare diseases? n/N (%) agree			
Total cohort	8/44 (18%)		
Academia	2/11 (18%)	RR:1.0 (95% CI = .24, 4.25), *p* = 1.0	
Private sector	6/33 (18%)		
Q: Do you agree that the current public information regarding reimbursement decisions in Australia provides sufficient information about which sources of value are considered and how they contributed to decision making, n/N (%)			
Total cohort	11/41 (27%) agree	Reasons for disagreement 6/30 (20%) state that we do not know which sources of value are considered 22/30 (73%) state that while we know which sources of value are considered, we do not know how they contribute to decision making 2/30 (6.7%) did not select a reason for disagreement	
Academia	5/11(45%) agree	RR: 2.27 (95% CI = .87, 5.97), *p* = .10	Reasons for disagreement – 2/6 (33%) state that we do not know which sources of value are considered – 2/6 (33%) state that while we know which sources of value are considered, we do not know how they contribute to decision-making – 2/6 (33%) not sure
Private sector	6/30 (20%) agree		Reasons for disagreement – 4/24 (17%) state that we do not know which sources of value are considered – 20/24 (83%) state that while we know which sources of value are considered, we do not know how they contribute to decision-making
Q: Do you agree that an explicit checklist of sources of value beyond the patient QALY and whether they were considered by the decision maker would be more informative than what is currently published in Australia? n/N (%) agree			
Total cohort	28/40 (70%)		
Academia	5/10 (50%)	RR: 0.65 (95% CI = .34, 1.25), *p* = .11	
Private sector	23/30 (77%)		
Q: Do you agree with the following mechanism, that MEAs should be used in Australia in making decisions about reimbursement of medicines for rare disease? n/N (%) agree			
Total cohort	32/40 (80%)		
Academia	9/10 (90%)	RR: 1.17 (95% CI = .88, 1.56), *p* = .36	
Private sector	23/30 (77%)		
Q: Do you agree with the following mechanism, that RSAs should be used in Australia in making decisions about reimbursement of medicines for rare disease, n/N (%)			
Total cohort	33/40 (83%)		
Academia	9/10 (90%)	RR: 1.13 (95% CI = .86, 1.48), *p* = .47	
Private sector	24/30 (80%)		
Q: Do you agree with the following mechanism, that MCDAs should be used in Australia in making decisions about reimbursement of medicines for rare disease? n/N (%)			
Total cohort	29/40 (73%)		
Academia	7/10 (70%)	RR: 0.96 (95% CI = .60, 1.51), *p* = .84	
Private sector	22/30 (73%)		
Q: Do you agree with the following mechanism, that WTP should be used in Australia in making decisions about reimbursement of medicines for rare disease? n/N (%)			
Total cohort	27/40 (68%)		
Academia	4/10 (40%)	RR: 0.52 (95% CI = .24, 1.14), *p* = .03	
Private sector	23/30 (77%)		

Abbreviations: CI, confidence interval; MCDA, multi-criteria decision analysis (involves a deliberative process where decision makers and stakeholders come together to define the problem and determine the criteria, weighting, and evidence requirements for decision (24;27)); MEA, outcome-based managed entry agreement (allows earlier market access but requires CEA review once additional outcome data are available. For example, clinical data from prespecified study protocol for all patients subsidized or from existing planned or progressing studies (26)); RR, relative risk; RSA, financial risk-sharing arrangement (with subsidy based on medicine or patient performance. For example, percentage rebate if the number of treatments per patient or the accepted duration of treatment is exceeded. Subsidy ceases if patients do not meet agreed clinical measures (26); WTP, willingness to pay (an increase in the ICER considered acceptable for treatments of rare diseases).

The majority of stakeholders (70 percent; academic = 50 percent vs. private sector = 77 percent, *p* = .1110) agreed that having an explicit checklist of additional value considered beyond the QALY by decision makers would be more informative than what is currently published in Australia (Table 3). There is an imbalance in responses; however, an RR of 0.65 suggests that the academic group was 35 percent less likely to agree than private sector stakeholders that a checklist may be more informative. Importantly, the 95 percent CI (range = .34–1.25) indicates uncertain precision of this effect.

Stakeholders were invited to explore mechanisms to facilitate expedited access to treatments for rare diseases while effectively managing the uncertainties associated with CE analysis (CEA) and budgetary impacts (BIs). This consideration is driven by the significant unmet need and the demand for accelerated access to such medicines.

Most Australian stakeholders (>68 percent) agreed that the four mechanisms (MEAs, financial risk-sharing arrangements (RSAs), MCDAs, and increased ICERs considered acceptable for treatments of rare diseases denoted as willingness to pay (WTP)) should be used in making reimbursement decisions about medicines for rare diseases (Table 3). Over 70 percent of academia and private sector respondents agreed that MEAs, RSAs, and MCDAs should be used in making reimbursement decisions for medicines for rare diseases in Australia (Table 3). Significantly fewer academic respondents (40 percent) compared with the majority of private sector respondents (77 percent) (*p* = .0320) agreed that an increase in the ICERs considered acceptable for medicines for rare diseases in Australia should be used in decision making.

The majority of all Australian stakeholders (>65 percent) believed that 6 of the 11 broader value elements recommended by ISPOR, that is, labor productivity, adherence, reducing uncertainty due to a new diagnostic, severity of disease, value to caregivers, and equity, should be considered in HTA of all medicines, including those for rare diseases (Table 4). In contrast, few stakeholders agreed that the value of hope, real option value, scientific spillover, fear of contagion, or insurance value should be considered (Table 4).

**Table 4.** Comparison between sources of value that should be considered in HTA for all medicines, including those for rare diseases

Broad elements of value	All medicines, *n*/*N* (%) agree				Medicines for rare disease, *n*/*N* (%) agree			
	Total cohort	Stakeholder sectors			Total cohort	Stakeholder sectors		
		Academia	Private sector	RR (95% CI), *p*-value		Academia	Private sector	RR (95% CI), *p*-value
Labor productivity	31/43 (72%)	6/11 (55%)	25/32 (78%)	.70 (.40, 1.23), .1326	26/40 (65%)	5/10 (50%)	21/30 (70%)	.71 (.37, 1.39), .2508
Adherence	39/42 (93%)	9/10 (90%)	30/32 (94%)	.96 (.77, 1.20), .6877	34/40 (85%)	8/10 (80%)	26/30 (87%)	.92 (.66, 1.30), .6091
Reducing uncertainty due to a new diagnostic	37/41 (90%)	8/10 (80%)	29/31 (94%)	.86 (.62, 1.18), .2093	34/40 (85%)	7/10 (70%)	27/30 (90%)	.78 (.51, 1.19), .1250
Fear of contagion	6/41 (15%)	1/10 (10%)	5/31 (16%)	.62 (.08, 4.70), .6335	4/40 (10%)	1/10 (10%)	3/30 (10%)	1.0 (.12,8.56), 1.00
Insurance value	4/41 (10%)	0	4/31 (13%)	ND	6/40 (15%)	1/10 (10%)	5/30 (17%)	.60 (.08, 4.54), .6091
Severity of disease	30/41 (73%)	8/10 (80%)	22/31 (71%)	1.13 (.77, 1.65), .5751	31/40 (78%)	8/10 (80%)	23/30 (77%)	1.04 (.73, 1.51), .8270
Value of hope	15/40 (38%)	2/10 (20%)	13/30 (43%)	.46 (.13, 1.70), .1869	9/40 (22%)	1/10 (10%)	8/30 (27%)	.38 (.05, 2.64), .2744
Value to caregivers	36/40 (90%)	8/10 (80%)	28/30 (93%)	.86 (.62, 1.19), .2235	35/40 (88%)	7/10 (70%)	28/30 (93%)	.75 (.50, 1.14), .0533
Real option value	17/40 (43%)	3/10 (30%)	14/30 (47%)	.64 (.23, 1.78), .3558	11/40 (28%)	0	11/30 (37%)	ND
Scientific spillover	11/40 (28%)	2/10 (20%)	9/30 (30%)	.67 (.17, 2.58), .5397	10/40 (25%)	1/10 (10%)	9/30 (30%)	.33 (.05, 2.32), .2059
Equity	32/40 (80%)	7/10 (70%)	25/30 (83%)	.84 (.54, 1.30), .3613	34/40 (85%)	8/10 (80%)	26/30 (87%)	.92 (.66, 1.30), .6091

Note: The questions posed to participants were: “Rate the extent to which you agree or disagree that the following source of value should be considered in HTA of medicines in Australia,” and then participants nominated if they were aware of methods to include each source of value in a cost-effectiveness analysis (presented in Table 5); a table of 11 value elements was then presented to participants and they were asked “Do you agree that the following sources of value should be considered in cost-effectiveness analysis of a medicine for rare diseases in Australia?” for each value.

Abbreviations: CI, confidence interval; ND, not determined; RR, relative risk.

The degree of consensus between the stakeholder groups is demonstrated by an RR close to 1.0, accompanied by a narrow CI. This indicates a consensus between academia and private sector respondents on the majority of broader value elements agreed should be considered in HTA of all medicines, including those for rare diseases in Australia, namely adherence, reducing uncertainty due to a new diagnostic, severity of disease, and equity. Furthermore, the analysis revealed no statistically significant differences in responses when analyzed by sector (Table 4). Interestingly, the likelihood of agreeing to include “labor productivity” in HTA of all medicines, including those for rare diseases in Australia, is ~30 percent less in the academic respondents compared with private sector respondents. Also, the likelihood of the academic group agreeing to include “Value to caregivers” in HTA of medicines for rare diseases in Australia (RR = 0.75) is 25 percent less likely than the private sector; yet, the degree of concordance was greater when considering HTA of all medicines (RR = 0.86). In addition, the majority of both stakeholder groups did not agree that the “Value of hope” should be considered in HTA of all medicines, including those for rare diseases in Australia (<43 percent); the likelihood of agreeing that it should be included was 50 percent lower in the academic respondents compared with private sector respondents (RR = 0.46 and RR = 0.38, respectively). Each group was aware of methods to capture impacts on costs and outcomes across broader sources of value, such as QoL measures, subgroup analysis, and distributional CEA (DCEA) (Table 5).

**Table 5.**
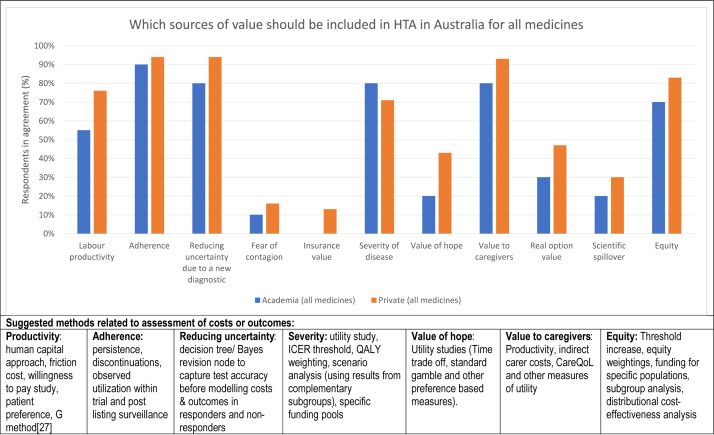
Comparison between stakeholder groups regarding sources of value that should be considered in HTA for all medicines and suggested methods to include them into EEs

## Discussion

This study examined the views from academic and private sector stakeholders involved in HTA on the broad elements of value that should be considered by decision makers in Australia, as well as mechanisms to mitigate uncertain CE and BI associated with medicines for rare diseases.

The majority of respondents agreed that current public information regarding reimbursement decisions in Australia provides insufficient information about the consideration of sources of value in decision making. Furthermore, the majority of respondents agreed that the current HTA methods applied in Australia are inadequate for appropriately assessing the CE of all medicines, including those for rare diseases. Australian reimbursement recommendations are made transparent to the public by publishing them online as public summary documents (PSDs) (29). They provide contextual information pertaining to each recommendation, and although they are limited in terms of the amount of information published, they provide insight into the factors and trade-offs noted through the deliberative process in arriving at reimbursement recommendations (30). Transparency on which inputs are accepted (and under what conditions) by HTA decision makers is necessary because it enables stakeholders to collect relevant data to inform decision making (31). This study highlights transparency on what was considered in PBAC decision making in the PSD, which needs further improvement. Of interest, participants in Australia’s recent HTA policies and methods review (referred to as the “HTA review”) expressed concern that PSDs fail to adequately convey how certain evidence types impact health technology funding decisions (27). The HTA review findings are consistent with those from this survey.

More private sector stakeholders (77 percent) than academic stakeholders (50 percent) thought an explicit checklist of sources of value beyond the QALY, and information on whether they were considered by a decision maker, would be more informative than what is currently published in Australia. Private sector stakeholders, particularly those in the pharmaceutical industry, may have more interest in PBAC decision making than academics, as they depend on these decisions for medication funding (Table 1). Nonetheless, experts suggest a checklist for reimbursement decision making as a useful framework to standardize the consideration of sources of value, minimize bias, and improve transparency (7;9;18;32). The HTA review recommends that the Australian Government develop and support an explicit qualitative values framework to ensure HTA decisions consider broader value, enhancing transparency and consistency in funding health technologies (27). Importantly, the recommendation states that the framework should allow enough flexibility for the deliberation process itself to add value that is not preweighted and scored. Examples of explicit qualitative value frameworks and transparent reporting by HTA committees include the Institute for Clinical and Economic Review (I.C.E.R.) in the United States that refers to “potential other benefits and contextual considerations,” such as health disparities, caregiver burden, or impact the entire “infrastructure” of care that committee members individually rate during deliberation (28). The I.C.E.R. value framework is systematic regarding the factors incorporated into decision making and explicitly reported (29). NICE includes nonquantified additional health benefits, such as contributions to the health system (e.g., equity), and innovation (25). The final outcome of NICE describes how such “other factors” impacted decision making (30).

Less than 20 percent of Australian stakeholders agree that fear of contagion and insurance value should be considered in the HTA of all medicines, including those for rare diseases in Australia. Six broad value elements that most Australian stakeholder felt should be considered in the HTA of medicines in Australia (labor productivity, adherence, reducing uncertainty due to a new diagnostic, severity of disease, value to caregivers, and equity) are recommended in several HTA guidelines, whereas only two (severity of disease and equity) overlap with the “less readily quantifiable” factors quoted to “influence” PBAC decision making in Australia (5;33). The PBAC guidelines highlight several factors considered during PBAC deliberations, such as the overall confidence in the evidence and assumptions presented, equity, severity, the capacity to target therapy, the existence of effective therapeutic alternatives, public health considerations, and any other pertinent factor influencing a medicine’s suitability for listing on the PBS (Pharmaceutical Benefits Scheme). These qualitative assessments, along with CE and BI, may obscure the weight of each factor in reimbursement decisions. Additionally, while the guidelines assert that “Supplementary analyses may be appropriate where the proposed intervention has important societal implications” – thereby permitting the inclusion of broader values in supplementary CEA – the relegation of nonhealth benefits to supplementary analyses might result in them being overlooked in the decision-making process and omitted from the PSD.

A recent review of fifty-three HTA guidelines representing fifty-two countries found an average of 5.9 out of a possible twenty-one societal and novel value elements were mentioned; however, the authors acknowledge that simply recommending novel elements of value in HTA guidelines may not lead to them being incorporated into decision-making processes (5). The Australian HTA guidelines outline a preferred approach for PBAC submissions but allow alternative approaches if justified with data. Stakeholders can include alternative value elements in their submissions, but decision makers must transparently evaluate these. Transparency is crucial for sponsors, as developing evidence is a resource-intensive and can guide future evidence generation. Including well-supported, broader value elements in decision making acknowledges therapy benefits and aids patient access to medicines (15;16;19).

There are challenges in quantifying some broad value elements, and a lack of consistent methodology for their inclusion in EEs, as well as a lack of expertise in assessing the methodological approaches (5;8;9). Both stakeholder groups were generally aware of methods to incorporate agreed-upon value elements into the HTA of medicines. However, some elements, like fear of contagion and insurance value, lacked acknowledged methods. Suggested methods, within the CE framework, included preference-based methods, scenario analysis, and DCEA. Academics had higher method knowledge, indicating varying skill sets among stakeholders. This underscores the need for PBAC guidelines to guide data and methods to support broader value elements, alongside improving transparency in decision making. For example, the Medical Services Advisory Committee in Australia includes the “value of knowing” as a less quantifiable factor influencing decisions and offers technical guidance on evidence to support this element (34).

The inquiry into proposed decision-making mechanisms for reimbursing medicines for rare diseases in Australia was framed by the context that medicines for rare diseases are generally expensive, with limited evidence of clinical effectiveness, attributed to small, noncomparative clinical studies and a lack of epidemiological data. RSAs and outcome-based MEAs are existing mechanisms employed in Australia to subsidize medicines despite the lack of confidence in the evidence for a medicine (26). Most stakeholders agreed that RSAs and MEAs should be used in making reimbursement decisions about medicines for rare diseases. RSAs, described in this study, are a practical financial arrangement that continues to subsidize a medicine only when treated patients meet specific clinical criteria. It also provides certainty around financial expenditure to the government despite patient population size uncertainty. Outcome-based MEAs are challenging to implement in Australia due to the absence of infrastructure linking medicine utilization and clinical outcomes, and thus most MEAs implemented in Australia to date are limited to reviewing the recommendation to reimburse a medicine once additional outcome data becomes available from a clinical trial that is underway (26;35;36). If MEAs are to be used to expedite access to medicines for rare diseases in Australia despite uncertain clinical evidence, handling challenges such as establishing infrastructure to support comprehensive data collection as well as price adjustments based on outcomes arrangements or product delisting due to suboptimal performance are some of the significant tasks for both payers and the pharmaceutical industry (26;37).

The MCDA method referred to in this survey was a quantitative MCDA whereby stakeholder preferences are used to specify a value for each criterion, the values are weighted, and an overall score is generated for each intervention (21). The use of quantitative MCDA in HTA is not widespread; however, most Australian stakeholders responding to the survey believe it should be used to make reimbursement decisions about medicines for rare diseases in Australia (31). The formal structure of MCDA avoids some of the issues in less structured deliberative processes, explicitly elicits decision makers’ preferences, and allows for the inclusion of broader value elements important to stakeholders but not easily accommodated in standard CEAs (21;24;38). Two systematic reviews of quantitative MCDA found it useful for focusing discussion and reporting decisions transparently, but found no evidence of improved decision-making quality or timeliness (32;33). Importantly, the weighting of the relative importance of various value elements would likely differ between stakeholders such as patients and payers (21). Consequently, the HTA review recommendation is to develop a “qualitative value framework” that is neither preweighted nor prescored.

There was significant disagreement between the stakeholder groups regarding increasing WTP thresholds in making reimbursement decisions about medicines for rare diseases. Among the many countries that use CEs to inform funding decisions (such as England and Wales, Australia, New Zealand, Canada, Sweden, the Netherlands, and others), only England and Wales, as well as the Netherlands, use an explicit WTP threshold to make funding recommendations (39). The PBAC does not explicitly report a fixed WTP value to judge the acceptability of a medicine as CE; however, revealed and stated preference studies of PBAC decision making show a preference for smaller ICERs to recommend a medicine (40;41). The view from academic stakeholders aligns with surveys of the Australian general public, which shows there is no WTP for a premium for rarity, although there is a case for paying more for drugs that treat severe conditions, or where there is no alternative treatment available (42–44). Nonetheless, the PBAC has stated its willingness to accept a higher ICER in the face of significant uncertainty in the CE of a medicine for a rare disease (35).

A limitation of our study is the small sample size in this survey, as well as the unequal group sizes (academia, *N* = 11 and private sector, *N* = 33). The timing of the survey, conducted during the recent HTA review in Australia, may have influenced participation, as stakeholders could have experienced fatigue due to the extensive feedback collection during the review. Discrepancies in sample sizes may account for the lack of significant differences observed, as smaller samples increase variability and standard error, thereby reducing the reliability of estimates and the sensitivity to detect differences. Additionally, recruitment through email and professional societies may introduce selection bias, as it depends on self-selection by more engaged stakeholders. Nevertheless, the participants had considerable expertise, averaging between 7 and 14 years of HTA experience, predominantly with health economic qualifications (Table 1), making their opinions likely a reliable reflection of other health economists in Australia. The absence of data from critical groups, such as government policy makers and patient representatives, limits the generalizability of our findings. Further research to include insights from these groups and expand the sample size would be beneficial. There may be other value elements that stakeholders think should be considered in the HTA of medicines in Australia beyond what was considered in this survey. Nevertheless, the broad value elements in the survey cover a wide range of values from societal elements (health impacts beyond the treated individual and costs beyond the healthcare sector, such as productivity and scientific spillover) to novel elements (e.g., insurance value, fear of contagion, and the value of hope). Regardless, the list of broad elements of value is not intended to be the final preferences of stakeholders.

## Conclusion

The perspectives of Australian stakeholders in both the academic and private sectors were largely congruent, showing no statistically significant differences in their views about the adequacy of current methods to assess the CE of all medicines, including those for rare diseases. Stakeholders from both sectors involved in HTA in Australia expressed concerns that current HTA methods in Australia are inadequate for assessing the broader value of medicines. The private sector was particularly concerned that public statements about the funding of medicines lack transparency regarding which specific value sources influenced reimbursement decisions.

There was consensus among both groups favoring the inclusion of more value elements in HTA decision making than currently recognized in the PBAC guidelines, specifically advocating for the integration of 6 out of the 11 values from the ISPOR value framework.

The survey’s findings offer valuable insights relevant to the Australian HTA review’s recommendations, suggesting that an explicit qualitative framework be developed, informed by public consultation and existing research. Additional research to gather perspectives from patients and decision makers and to increase the sample size would be advantageous. This study underscores the necessity for enhanced guidance in reimbursement guidelines and for greater transparency in the publication of decisions related to the values influencing decision making.

## Supporting information

Farris et al. supplementary material Farris et al. supplementary material

## Data Availability

Data used in this study can be shared upon reasonable request to the corresponding author.

## Supplementary material

The supplementary material for this article can be found at http://doi.org/10.1017/S0266462325100226.
