# Supplementary material for: Stakeholder survey about broad elements of value in health technology assessment in Australia: industry and academia more similar than different
Source: Int J Technol Assess Health Care. 2025 Jul 8;41(1):e61. doi: 10.1017/S0266462325100226 (PMC12390742; doi:10.1017/S0266462325100226)
Supplement: Farris et al. supplementary material [file S0266462325100226sup001.pdf]

Default Question Block

[UTS HREC REF NO. ETH21-6090] – On-line stakeholder survey: Consideration of broader value in health technology assessment (HTA) in Australia-PARTICIPANT INFORMATION SHEET

WHO IS CONDUCTING THIS RESEARCH?

My name is Maria (preferred name Mish) Farris (Maria.H.Kokoszk@alumni.uts.edu.au) and I am a research student at the Centre for Health Economics Research and Evaluation (CHERE), part of the Faculty of Health at the University of Technology Sydney (UTS). I am conducting the research under an Industry Doctorate Program with UTS as an employee of AstraZeneca. My supervisors are Professor Stephen Goodall and Associate Professor Richard De Abreu Lourenco and they can be contacted at Stephen.Goodall@uts.edu.au and Richard.DeAbreuLourenco@uts.edu.au.

Your participation in this research is totally voluntary, and information is provided below to help you to decide if you wish to participate. Contact details are also provided, for any questions you may have.

WHAT IS THE RESEARCH ABOUT?

Health technology assessment (HTA) systematically examines the comparative evidence of safety, clinical efficacy and cost-effectiveness of medicines to support reimbursement (funding allocation). This research is to understand the views held by stakeholders involved in HTA in Australia on the consideration of sources of value beyond the QALY in economic evaluations of medicines, particularly for rare diseases.

To participate in this research, you don't need to have a prior understanding of sources of value beyond the QALY; a

description of other potential sources of value is provided in the survey.

The information you provide will be used to understand views from different stakeholders such as the private sector (pharmaceutical industry, specialist consultants and executive members of patient organisations), as well as public sector stakeholders (academia and government) about the role for different sources of value in HTA. The survey is anonymous and we would like you to be completely open with your views which will be treated in total confidence.

WHY HAVE I BEEN INVITED?

You are being asked to participate because you are a stakeholder involved in HTA in Australia.

Before you decide to participate in this research study, please confirm that the following apply to you:

- *Are an adult (age ≥18) resident of Australia, and willing to give explicit informed consent to participate and for answers to be recorded.*
- *Are involved in HTA in Australia as a member of the pharmaceutical industry, specialist consultants, executive members of patient organisations, HTA evaluators, academic, reimbursement committee or Government.*
- *Able to complete the survey via a web-based tool (Qualtrics).*

FUNDING

There is no remuneration for completing the survey. MF has received funding support for this project from AstraZeneca Australia, Pty Ltd.

WHAT DOES MY PARTICIPATION INVOLVE?

If you decide to participate, you will be presented with 24 questions with a selection of responses to choose from. You will be asked to answer some background questions such as type of organisation, position in the organisation, length of time involved in HTA and academic qualifications. You will be asked to nominate whether you agree or disagree with various statements and you will also be asked to rate how much you agree with statements that are presented to you.

The survey is presented on-line via the Qualtrics platform. The survey is expected to take 10-20 minutes to complete.

ARE THERE ANY RISKS/INCONVENIENCE?

We do not anticipate any health risks to you from participating in this study. The survey is anonymous meaning we will not collect personal details such as your name or any identifying characteristics.

We do not anticipate that any of the questions included in the survey will cause discomfort to you. There will be no

direct health benefit to you from participating in this study.

DO I HAVE TO TAKE PART IN THIS RESEARCH PROJECT?

Your participation in this research is totally voluntary and choosing to participate or declining to participate will not impact your relationship with researchers or the University of Technology Sydney. You can decline to participate for any reason, and do not need to justify your decision. You are fully able to decline to participate, or decline to continue to participate, now or at any other time. If you decline to participate, we will not capture any more information from you.

WHAT IF I WITHDRAW FROM THIS RESEARCH PROJECT?

If you wish to withdraw from the survey once it has started, you can do so at any time without having to give a reason. If you do decide to withdraw from the study while completing the survey, it may not be possible to withdraw your data from the study results. This is because your responses are recorded as you progress through the survey. If you are interrupted during the survey and are unable to finish you will have 1 week to come back to the survey to complete the survey, otherwise your partial response is recorded as complete.

WHAT WILL HAPPEN TO INFORMATION ABOUT ME?

By providing your consent, you agree to the research team collecting and using background information about you for the research project and your survey responses. All this information will be anonymous because your data will not be linked to any personal identifiers. The Qualtrics database will not collect your IP address, location data or contact information.

Your background information will only be used for the purpose of this research project.

It is anticipated that the results of this research project will be published and/or presented in a variety of forums. In any publication and/or presentation, information will be provided in such a way that you cannot be identified, except with your permission.

The results of this research may also be shared through open access (public) scientific databases, including internet databases. This will enable other researchers to use the data to investigate other important research questions.

WHAT IF I HAVE ANY QUERIES OR CONCERNS?

If you have queries or concerns about the research that you think I or my supervisor can help you with, please feel free to contact us via one of the following email addresses Maria.H.Kokoszk@alumni.uts.edu.au, Stephen.Goodall@uts.edu.au or Richard.DeAbreuLourenco@uts.edu.au. Alternatively you can contact me on ph: +61 2 9514 4720.

NOTE:

This study has been approved in line with the University of Technology Sydney Human Research Ethics Committee [UTS HREC] guidelines. If you have any concerns or complaints about any aspect of the conduct of this research that you wish to raise independently of the research team, please contact the Ethics Secretariat on ph.: +61 2 9514 2478 or email: Research.Ethics@uts.edu.au], and quote the UTS HREC reference number. Any matter raised will be treated confidentially, investigated and you will be informed of the outcome.

[UTS HREC REF NO. ETH21-6090] – On-line stakeholder survey: Consideration of broader value in health technology assessment (HTA) in Australia-CONSENT FORM

I agree to participate in the research project being conducted by Maria (Mish) Farris [Maria.H.Kokoszk@alumni.uts.edu.au or ph:+61 2 9514 4720].

I have read the Participant Information Sheet or someone has read it to me in language that I understand.

I understand the purposes, procedures and risks of the research as described in the Participant Information Sheet.

I freely agree to participate in this research project as described and understand that I am free to withdraw at any time without affecting my relationship with the researchers.

I am aware that I can contact Maria Farris, Professor Stephen Goodall, Associate Professor Richard De Abreu Lourenco or the Ethics Secretariat if I have any concerns about the research.

Please check the following box to indicate your consent

- ○ I give consent
- ○ I do not give consent

Block 45

Please select which applies to you

- ○ Pharmaceutical Industry
- ○ Consultants
- ○ Academia
- ○ Government Agency
- ○ Representative of patient organisation

○ [ ] Other (please specify)

How many years have you been involved in Health Technology Assessment (HTA) in Australia for?

[ ]

Block 2

Please nominate your position in your organization

○ Managerial role
○ Non-managerial role

Block 3

Academic qualification

○ Doctoral degree
○ Masters degree
○ Undergraduate degree
○ No degree

Block 4

Area of academic qualification

- ☐ Science
- ☐ Pharmacy
- ☐ Health economics
- ☐ Statistics
- ☐ Medicine
- ☐ [ ] Other (please specify)
- ☐ None

Block 5

Do you think the current HTA methods applied in Australia are adequate to appropriately assess the cost effectiveness of **all medicines?**

- ○ Yes
- ○ No
- ○ Not sure

Block 6

Do you think the current HTA methods applied in Australia are adequate to appropriately assess the cost effectiveness of **medicines for rare diseases?**

- ○ Yes
- ○ No
- ○ Not sure

Block 14

In some HTA markets, sources of value beyond the patient QALY are taken into account in a reimbursement decision. An ISPOR task force defined 11 possible additional elements of value to consider.

Block 18

Rate the extent to which you agree or disagree that the following source of value should be considered in HTA of medicines in Australia.

	Strongly agree	Somewhat agree	Neither agree nor disagree	Somewhat disagree	Strongly disagree
1.Labour Productivity: Relates to costs associated with production loss and replacement costs due to illness, disability and death of productive persons, both paid and unpaid.	○	○	○	○	○

Block 24

Are you aware of methods to include Labour Productivity in a cost effectiveness analysis?

- ○ Yes
- ○ No
- ○ Not sure

Block 19

Rate the extent to which you agree or disagree that the following source of value should be considered in HTA of medicines in Australia?

	Strongly agree	Somewhat agree	Neither agree nor disagree	Somewhat disagree	Strongly disagree
2.Adherence: Patient adherence and health outcomes relating to advantageous simpler dosing schedules, alternate routes of administration, or combination treatments over existing alternatives.	○	○	○	○	○

Block 29

Are you aware of methods to include Adherence in a cost effectiveness analysis?

- ○ Yes
- ○ No
- ○ Not sure

Block 41

Please nominate whether these methods relate to costs and/or outcomes below by ticking all that apply? Please name the method in the free text field if you are able to.

☐ Outcomes

☐ Costs

☐ [ ] Name of the method

Block 20

Rate the extent to which you agree or disagree that the following source of value should be considered in HTA of medicines in Australia?

	Strongly agree	Somewhat agree	Neither agree nor disagree	Somewhat disagree	Strongly disagree
3.Reducing uncertainty due to a new diagnostic: A companion diagnostic test that could differentiate “good responders” and “poor responders” may provide the ability to avoid an ineffective treatment in poor responders as well as costs and consequences of treatment-related adverse events.	○	○	○	○	○

Block 32

Are you aware of methods to include Reducing uncertainty due to a companion new diagnostic in a cost effectiveness analysis?

- ◯ Yes
- ◯ No
- ◯ Not sure

Block 41

Please nominate whether these methods relate to costs and/or outcomes below by ticking all that apply? Please name the method in the free text field if you are able to.

- ☐ Outcomes
- ☐ Costs
- ☐ [ ] Name of the method

Block 21

Rate the extent to which you agree or disagree that the following source of value should be considered in HTA of

medicines in Australia?

	Strongly agree	Somewhat agree	Neither agree nor disagree	Somewhat disagree	Strongly disagree
4. Fear of contagion: Reducing the anxiety associated with the risk of future illness, even if the expected number of cases prevented is low.	○	○	○	○	○

Block 33

Are you aware of methods to include Fear of Contagion in a cost effectiveness analysis?

- ○ Yes
- ○ No
- ○ Not sure

Block 42

Please nominate whether these methods relate to costs and/or outcomes below by ticking all that apply? Please name the method in the free text field if you are able to.

☐ Outcomes

☐ Costs

☐ [ ] Name of the method

Block 23

Rate the extent to which you agree or disagree that the following source of value should be considered in HTA of medicines in Australia?

	Strongly agree	Somewhat agree	Neither agree nor disagree	Somewhat disagree	Strongly disagree
5.Insurance value: reflects the value from an effective treatment for a disease reducing fear among all consumers of getting the disease.	○	○	○	○	○

Block 34

Are you aware of methods to include Insurance Value in a cost effectiveness analysis?

○ Yes

○ No
○ Not sure

Block 43

Please nominate whether these methods relate to costs and/or outcomes below by ticking all that apply? Please name the method in the free text field if you are able to.

- ☐ Outcomes
- ☐ Costs
- ☐ [ ] Name of the method

Block 22

Rate the extent to which you agree or disagree that the following source of value should be considered in HTA of medicines in Australia?

	Strongly agree	Somewhat agree	Neither agree nor disagree	Somewhat disagree	Strongly disagree
6.Severity of disease: A gain in health may be more valuable to patients with a poor baseline prognosis (i.e., more severe disease).	○	○	○	○	○

Block 35

Are you aware of methods to include Severity of Disease in a cost effectiveness analysis?

- ○ Yes
- ○ No
- ○ Not sure

Block 44

Please nominate whether these methods relate to costs and/or outcomes below by ticking all that apply? Please name the method in the free text field if you are able to.

- ☐ Outcomes

- [ ] Costs
- [ ] [ ] Name of the method

Block 29

Rate the extent to which you agree or disagree that the following source of value should be considered in HTA of medicines in Australia?

	Strongly agree	Somewhat agree	Neither agree nor disagree	Somewhat disagree	Strongly disagree
7.Value of hope: Reflects the extent to which the chance for a cure is valued. For example, some patients may be willing to trade some survival (e.g. undertake a risky procedure) for a chance of a "cure" even if only for a small probability of cure/improved survival.	○	○	○	○	○

Block 36

Are you aware of methods to include Value of Hope in a

cost effectiveness analysis?

- ○ Yes
- ○ No
- ○ Not sure

Block 45

Please nominate whether these methods relate to costs and/or outcomes below by ticking all that apply? Please name the method in the free text field if you are able to.

- ☐ Outcomes
- ☐ Costs
- ☐ [ ] Name of the method

Block 28

Rate the extent to which you agree or disagree that the following source of value should be considered in HTA of medicines in Australia?

	Strongly agree	Somewhat agree	Neither agree nor disagree	Somewhat disagree	Strongly disagree
8.Value to caregiver: Extent to which health care can benefit family carers by reducing their caring responsibilities.	○	○	○	○	○

Block 37

Are you aware of methods to include Value to Caregiver in a cost effectiveness analysis?

- ○ Yes
- ○ No
- ○ Not sure

Block 46

Please nominate whether these methods relate to costs and/or outcomes below by ticking all that apply? Please name the method in the free text field if you are able to.

- ☐ Outcomes
- ☐ Costs

☐ [ ] Name of the method

Block 27

Rate the extent to which you agree or disagree that the following source of value should be considered in HTA of medicines in Australia?

	Strongly agree	Somewhat agree	Neither agree nor disagree	Somewhat disagree	Strongly disagree
9.Real option value: Value generated when a health technology that extends life creates opportunities for the patient to benefit from other future advances in medicine.	○	○	○	○	○

Block 38

Are you aware of methods to include Real Option Value in a cost effectiveness analysis?

○ Yes

○ No

○ Not sure

Block 47

Please nominate whether these methods relate to costs and/or outcomes below by ticking all that apply? Please name the method in the free text field if you are able to.

☐ Outcomes

☐ Costs

☐ [ ] Name of the method

Block 26

Rate the extent to which you agree or disagree that the following source of value should be considered in HTA of medicines in Australia?

	Strongly agree	Somewhat agree	Neither agree nor disagree	Somewhat disagree	Strongly disagree
10.Scientific spillovers: Broad societal benefit from knowledge created from a treatment with a new mechanism of action. It is considered a public good used for the discovery of other agents.	○	○	○	○	○

Block 39

Are you aware of methods to include Scientific Spillover in a cost effectiveness analysis?

- ○ Yes
- ○ No
- ○ Not sure

Block 48

Please nominate whether these methods relate to costs and/or outcomes below by ticking all that apply? Please name the method in the free text field if you are able to.

- ☐ Outcomes

☐ Costs

☐ [ ] Name of the method

Block 25

Rate the extent to which you agree or disagree that the following source of value should be considered in HTA of medicines in Australia?

	Strongly agree	Somewhat agree	Neither agree nor disagree	Somewhat disagree	Strongly disagree
11.Equity: Fairness in the distribution of health and health care within society, across rich and poor, young and old, marginalized and not, employed or unemployed for example.	○	○	○	○	○

Block 40

Are you aware of methods to include Equity in a cost effectiveness analysis?

○ Yes

○ No

○ Not sure

Block 17

Please nominate whether these methods relate to costs and/or outcomes below by ticking all that apply? Please name the method in the free text field if you are able to.

☐ Outcomes

☐ Costs

☐ [ ] Name of the method

Block 8

Do you agree that the following sources of value should be considered in a cost effectiveness analysis of a medicine **for rare diseases in Australia?**

	Yes	No	Not sure
Labour productivity	○	○	○
Adherence	○	○	○

	Yes	No	Not sure
Reducing uncertainty due to a new diagnostic	○	○	○
Fear of contagion	○	○	○
Insurance value	○	○	○
Severity of disease	○	○	○
Value of hope	○	○	○
Value to caregiver	○	○	○
Real option value	○	○	○
Scientific spillover	○	○	○
Equity	○	○	○

Block 9

Do you agree that the current **public information regarding reimbursement decisions** in Australia provides sufficient information about which sources of value are considered and how they contributed to decision-making?

○ Yes

○ No

○ Not sure

Block 12

Please specify which of the following apply:

- ○ we don't know which sources of value are considered
- ○ while we know which sources of value are considered, we don't know how they contribute to decision-making

Block 10

Do you agree that an **explicit checklist** of sources of value beyond the patient QALY and whether they were considered by decision maker would be more informative than what is currently published in Australia?

- ○ Yes
- ○ No
- ○ Not sure

Block 13

Generally medicines for rare disease are expensive, the clinical evidence is from short term small single arm

studies and the estimated market size and expected utilisation is not known. This leads to greater uncertainty in cost-effectiveness analysis and budget impact which delays reimbursement decisions. However the high unmet need means there is a desire for accelerated reimbursement and delayed access could result in significantly higher costs and health losses. Thus some countries have implemented a range of mechanisms to enable faster access.

Block 11

Rate the extent to which you agree or disagree that the following mechanisms should be used in Australia in making decisions about the reimbursement of medicines **for rare disease**?

	Strongly agree	Somewhat agree	Neither agree nor disagree	Somewhat disagree	Strongly disagree
Multiple Criteria Decision Analysis (MCDA) a deliberative process where decision makers and stakeholders define the problem and determine the criteria, weighting and evidence requirements for a reimbursement decision.	○	○	○	○	○
	Strongly agree	Somewhat agree	Neither agree nor disagree	Somewhat disagree	Strongly disagree
Willingness to pay (WTP) Increase the ICER considered acceptable for treatments of rare diseases.	○	○	○	○	○
	Strongly agree	Somewhat agree	Neither agree nor disagree	Somewhat disagree	Strongly disagree

	Strongly agree	Somewhat agree	Neither agree nor disagree	Somewhat disagree	Strongly disagree
Outcome based Managed Entry Agreements (MEA) allows earlier market access but requires CEA review once additional outcome data are available. For example: clinical data from pre-specified study protocol for all patients subsidised or from existing planned or progressing studies.	○	○	○	○	○

	Strongly agree	Somewhat agree	Neither agree nor disagree	Somewhat disagree	Strongly disagree
Financial risk share arrangements (RSA) with subsidy based on medicine or patient performance. For example: Percentage rebate if the number of treatments per patient or accepted duration of treatment is exceeded. Subsidy ceases if patients do not meet agreed clinical measures.	○	○	○	○	○

Powered by Qualtrics
